# Impact of government pyrotechnics ban on emergency department usage rates around the turn of the years

**DOI:** 10.1007/s00068-025-03002-6

**Published:** 2025-11-14

**Authors:** Saskia Ehrentreich, Nele Kamer, Susanne Drynda, Ronny Otto, Felix Walcher, Benjamin Lucas

**Affiliations:** 1 Institute for Public Health in Acute Medicine, University Medicine Magdeburg, Leipziger Straße 44, 39120 Magdeburg, Germany; 2 Department of Trauma Surgery, University Hospital Magdeburg, Leipziger Str. 44, 39120 Magdeburg, Germany

**Keywords:** Emergency department, Firework, Injuries, Pandemic-related ban, New year’s eve, New year

## Abstract

**Purpose:**

Annual discussions concerning emergency department (ED) utilization during New Year due to avoidable emergencies and potential “pyrotechnic bans” often highlight media reports of severe injuries. However, limited data exist on the ED burden and specific injury patterns. This study aimed to investigate firework-related ED cases in Germany around the turn of the year owing to the pyrotechnic ban during the COVID-19 pandemic.

**Methods:**

Data from the AKTIN Emergency Department Data Registry were analysed between 2019 and 2023. Cases on New Year’s Eve (compared with Saturdays) and New Year (compared with Sundays) were averaged. The results were stratified by year and evaluated to determine the impact of pandemic-related pyrotechnics and gathering bans. We conducted a descriptive analysis of emergency department (ED) visits based on patient characteristics, length of stay (LOS), presenting complaints using the CEDIS-PCL and selected diagnosis-group (ICD-10). Statistical significance was assessed using chi-square or Fisher’s exact test (*p* < 0.05), and effect sizes were estimated using Cohen’s w and phi.

**Results:**

31 emergency departments participated in the data query, but three emergency departments had to be excluded due to duplicates in the dataset. Analysis of 134,763 cases from 28 EDs revealed fewer cases on New Year’s Eve and more on New Year than on Saturdays and Sundays, respectively, with no significant change in length of stay. During New Year, there was a higher incidence of skin-, otorhinolaryngologist-, and orthopedics/trauma-related complaints. A slight increase in head injury diagnoses (ICD-10 codes) was observed. The pandemic-related firework bans did not affect ED utilization or injury patterns.

**Conclusion:**

The firework ban during the COVID-19 pandemic did not lead to a relevant reduction in overall emergency department utilization or injury patterns around New Year. Case numbers showed consistent temporal peaks between midnight and early morning hours across all years, including during the ban. A slight increase in cases after the ban was lifted does not indicate a substantial change in ED burden.

## Introduction

Besides their environmental impacts [1], fireworks cause avoidable injuries due to improper use or unauthorized pyrotechnics. In Germany, most firework-related injuries occur around New Year’s Eve. Every year, the increased workload in emergency departments (ED) and the potential firework bans are debated. Injuries range from superficial abrasions, severe burns [2], hearing damage [3, 4], visual impairment [5–11], severe hand injury, and amputations [12–15]. While some cases can be treated on an outpatient basis, others require surgical intervention or are fatal [16–18]. However, previous studies [5, 15, 16, 19] indicate that young males are particularly affected by firework-related injuries at this time of year, with severe cases potentially leading to life-changing disabilities [16].

The president of the German Medical Association called for a nationwide ban on uncontrolled fireworks by the year 2022 [20]. Additionally, Van Ypern et al. [21] showed that homemade and self-ignited fireworks accounted the highest proportion of surgical interventions (around New Year’s Eve). Therefore, the Dutch Society of Plastic Surgery advocates a ban or stricter regulations on fireworks [12]. Other studies indicated that restricting access to fireworks reduces firework-related injuries [2, 9], whereas legalization has the opposite effect [8, 22].

However, the Federal Minister of the Interior rejected the general ban on fireworks [23]. Furthermore, the Association of the Pyrotechnic Industry (VPI) emphasizes that “there are no reliable national figures to support the assertion of an increased number of emergencies caused by fireworks on New Year’s Eve” [24]. The economic significance of firework sales is reflected in published revenue statistics [25].

Due to the strained hospital conditions during the COVID-19 pandemic, the German government prohibited the non-commercial sale of category F2 fireworks for New Year’s Eve 2020/2021 and 2021/2022 [26, 27]. Besides excessive alcohol consumption, concerns regarding injuries caused by explosions and the associated increase in ED visits remain. The decision to ban fireworks, along with an additional ban on contact and gatherings and partial curfews at the turn of the year, was intended to relieve pressure on hospitals.

Previous research by Winicki et al. [15] demonstrated that illegal pyrotechnics are most frequently linked to severe injuries necessitating hospital admission. Such injuries often result in longer treatment durations, as measured by length of stay (LOS). As LOS can serve as an indicator of treatment complexity [28], prolonged stays in the ED imply a higher workload per patient and indirectly reflect increased strain on ED resources.

Except for a few detailed studies in ophthalmology and otorhinolaryngology [3–5, 29], available data on ED workload and the impact of legislative changes on firework-related injuries remain limited. Existing studies are constrained by their focus on time, space, and/or specialties, making comparisons challenging [2, 10, 13, 16, 22].

In this study, we examined the impact of restrictions on legal firework access and pandemic-related regulations on the number of emergency treatments for pyrotechnic injuries around New Year’s Eve from 2019 to 2023. Specifically, we analysed the impact of a national firework ban on ED utilization and injury severity patterns.

We expect that the pyrotechnics ban during the COVID-19 pandemic leads to a significant decrease in case numbers to the ED, while increasing the severity of injuries and prolonging treatment times (due to the use of illegal fireworks).

## Materials and methods

### Patients, study setting, and data basis

This retrospective, multicentre study obtained data from 31 EDs across Germany, representing all three levels of emergency care as defined by the Federal Joint Committee: basic, extended, and comprehensive [30]. Data from electronic health records, based on the German Emergency Department Medical Record version 2015.1, were used, including demographic data (age, sex), transport, initial acuity assessment (triage), presenting complaints according to the Canadian Emergency Department Information System-Presenting Complaint List (CEDIS-PCL, version 3.0) [31], emergency admission diagnoses, time of admission and discharge, and disposition.

Anonymized treatment data were obtained from the AKTIN Emergency Department Data Registry. The AKTIN Emergency Department Data Registry is a decentralized and federated research infrastructure. The registry enables the reuse of electronic health data collected during routine care in the emergency department. This ensures data collection without additional effort on the part of the emergency department staff [32]. A data analysis request (Project ID 2023-002) was submitted and approved by the registry’s Data Use and Access Committee. Data analysis was conducted by the Trusted Data Analytics Center (TDAC). The AKTIN project was approved by the Ethics Committee of Otto von Guericke University, Medical Faculty, Magdeburg (160/15–23.11.2015).

## Inclusion and exclusion criteria and plausibility check

This study used data from an expanding registry, with additional EDs joining the AKTIN ED registry over the study period (Table 1). All patients receiving emergency care at participating EDs between 01/01/2019 and 02/28/2023 were requested. Hundred and eighty two patients were excluded from the analysis due to implausible ages (120–130 years). Additionally, three EDs (448,438 cases) were excluded from all analysis due to duplicates in the data warehouse.

**Table 1:** Patients’ characteristics per change of year in absolute numbers and percentages (For the categories of age and gender, the total number of cases represents (Line “Case numbers”) 100%. For transport, acuity assessment and discharge, the number of documented cases represents 100%)

Change of year	2019/2020	2020/2021	2021/2022	2022/2023
Case numbers	25,900	26,012	42,010	40,841
Emergency department	15	22	27	28
Age (years)				
0-20	4,419 (17.1%)	3,694 (14.2%)	6,586 (15.7%)	6,726 (16.5%)
21-40	6,806 (26.3%)	6,382 (24.5%)	11,620 (27.7%)	10,957 (26.8%)
41-60	5,664 (21.9%)	5,905 (22.7%)	9,097 (21.7%)	8,801 (21.5%)
61+	9,011 (34.8%)	10,031 (38.6%)	14,705 (35.0%)	14,355 (35.1%)
Sex				
Female	12,663 (48.9%)	12,796 (49.2%)	20,203 (48.1%)	19,424 (47.6%)
Male	13,232 (51.1%)	13,210 (50.8%)	21,766 (51.8%)	21,305 (52.2%)
Divers	5 (0.02%)	6 (0.02%)	41 (0.1%)	112 (0.3%)
Transport				
ground based	6,949 (34.6%)	8,488 (44.1%)	11,930 (39.3%)	11,163 (38.2%)
air based	34 (0.2%)	36 (0.2%)	113 (0.4%)	48 (0.2%)
without transport	13,108 (65.2%)	10,730 (55.7%)	18,329 (60.3%)	18,000 (61.6%)
Total	20,091 (100%)	19,257 (100%)	30,372 (100%)	29,211 (100%)
transport not documented	5,809	6,758	11,638	11,630
Acuity assessment (triage)				
Red - immediate	347 (1.5%)	441 (1.8%)	754 (1.9%)	665 (1.7%)
Orange - very urgent	2,408 (10.3%)	2,961 (12.3%)	5,155 (13.3%)	4,740 (12.3%)
Yellow - urgent	7,496 (32.0%)	8,641 (35.9%)	13,622 (35.0%)	13,020 (33.9%)
Green - standard	11,777 (50.3%)	10,637 (44.2%)	17,156 (44.1%)	17,727 (46.1%)
Blue - non-urgent	1,380 (5.9%)	1,397 (5.8%)	2,199 (5.7%)	2,281 (5.9%)
Total	23,408 (100%)	24,077 (100%)	38,886 (100%)	38,433 (100%)
Acuity assessment not documented	2,492	1,935	3,124	2,408
Disposition				
Discharge (non-admitted)	12,943 (66.5%)	11,304 (62.0%)	19,011 (67.5%)	20,982 (69.9%)
Hospital-admission (admitted)	6,511 (33.5%)	6,934 (38.0%)	9,172 (32.5%)	9,040 (30.1%)
Total	19,454 (100%)	18,238 (100%)	28,183 (100%)	30,022 (100%)
Disposition not documented	6,446	7,774	13,827	10,819

**Fig. 1:**
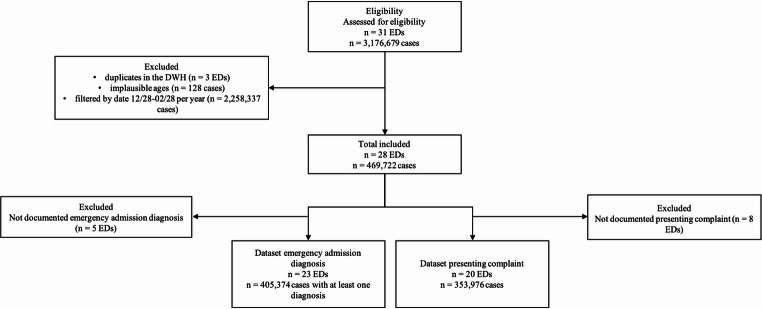
Flow chart illustrating inclusion and exclusion of emergency departments and included patients. (ED, emergency department; DWH, data warehouse)

For further analysis, eight EDs were excluded due to missing presenting complaints and five due to missing emergency admission diagnoses. For all analyses, treatment data from December 28 to February 28 were compared across the four years. These periods were considered annually for all variables (Fig. 1).

The identification of severe individual injuries in this study was limited by the coding system used for presenting complaints. Presenting complaints were recorded according to the Canadian Emergency Department Information System – Presenting Complaint List (CEDIS-PCL, version 3.0) [31], which is designed to capture a patient’s primary reason for visit in standardized categories. However, this system does not differentiate between severity levels of injuries within the same category. For example, a “head injury” may range from a minor laceration to a severe traumatic brain injury, but both would be assigned the same CEDIS-PCL code. Moreover, codes can be combined with other presenting complaints or diagnoses, depending on local documentation practices. As a result, severe injury cases cannot be reliably isolated solely on the basis of presenting complaint codes without additional clinical information.

### Statistical analyses

A descriptive analysis (mean or frequency) of the cohort was initially conducted for individual variables (age, sex, transport, acuity assessment, disposition, and presenting complaints according to the CEDIS-PCL). Cases per day (Saturday, New Year’s Eve; Sunday, New Year) and length of stay (LOS) were also analysed. LOS was calculated from admission to disposition timestamps, and cases with LOS > 24 h were excluded only for this analysis (519,239 cases). An ED LOS exceeding 24 h is generally considered implausible in routine emergency care and often indicates atypical circumstances or data quality issues (e.g., incorrect timestamps, administrative delays, extreme crowding). Therefore, LOS > 24 h was defined as a cut-off to identify and exclude outliers from analysis, ensuring validity and comparability of results. To better assess firework-related ED visits, we incorporated fireworks sales data [25].

Subsequently, patient subgroups admitted on New Year’s Eve and New Year were compared to control days (Saturdays and Sundays in the following eight weeks). The frequencies of presenting complaints from the major groups of skin, otorhinolaryngology, orthopaedics/trauma surgery, ophthalmology, substance abuse, and emergency admission diagnoses according to ICD-10, including injuries of the hands, head, and upper and lower extremities (S00-S99), were analysed. Statistical analyses were performed using the statistical program R (version 2023.06.2) [33].

Cross-tables and Pearson’s chi-square test were used to demonstrate statistical significance (*p* < 0.05). Due to the large number of cases, even small differences could be significant. Therefore, we used Cohen’s w to estimate the effect sizes: w 0.00 to < 0.1 (negligible effect), 0.1 to < 0.3 (small effect), 0.3 to < 0.5 (medium effect), and > 0.5 (large effect). For the 2 × 2 tables Fisher’s exact test and phi were used to assess effect sizes when significance was found (*p* < 0.05), with phi values ranging from 0 (no correlation) to 1 (perfect correlation).

## Results

### Demographic data

During the study period, data from 469,722 patients were analysed. Based on targeted search across the individual turns of the year and the restrictions on Saturdays, Sundays, New Year’s Eve, and New Year, we included 134,763 cases from 2019 to 2023: 69,513 males and 65,086 females (Table 1). Of the 15 included EDs in 2019/2020, 25,900 cases were registered on Saturdays and Sundays, as well as on New Year’s Eve and New Year. In 2020/2021, there were 22 included EDs with 26,012 cases; in 2021/2022, there were 27 EDs with a total of 42,010 cases; and in 2022/2023, there were 28 EDs with a total of 40,841 cases.

Most patients arrived at the emergency department without transport. The turn of year 2019/2020 represented the largest percentage (65.2%), followed by 2022/2023 (61.6%), 2021/2022 (60.3%), and 2020/2021 (55.7%).

For the analysis, we summarized the triage systems Manchester Triage System (MTS) and Emergency Severity Index (ESI). Seventeen EDs used MTS and 11 used ESI. As listed in Table 1, the frequencies of the highest triage level were comparable between 2019 and 2023 (1.5% vs. 1.8% vs. 1.9% vs. 1.7%).

More than half of the patients were treated as outpatients. The highest hospitalisation rate was in 2020/2021 (38.0%), followed by 2019/2020 (33.5%), 2021/2022 (32.5%), and 2022/2023 (30.1%).

### Firework sales data and caseload

Table 2 presents sale figures for each year relative to the number of cases. Firework sales were lowest during the pandemic years (2020/2021: €20 million; 2021/2022: €21 million) compared to 2019/2020 (€122 million) and 2022/2023 (€180 million), reflecting the ban on private fireworks [25].

**Table 2:** Nationwide sale figures, numbers of EDs and case numbers of included EDs per change of year (ED=Emergency Department, n=case numbers, SD= standard deviation; For the calculation of the deviations on New Year and New Year’s Eve, 100% are the respective case numbers of Saturdays and Sundays)

Change of year	Sale figures (million )	ED	n Saturday (SD;%)	n New Year’s Eve (SD;%)	n Sunday (SD;%)	n New Year (SD;%)
2019/2020	122	15	1,308 (± 127; 100%)	1,311 (100%)	1,258 (± 138.4; 100%)	1,485 (118%)
2020/2021	20	22	1,365 (± 98.7; 100%)	1,161 (85%)	1,235 (± 94.4; 100%)	1,445 (117%)
2021/2022	21	27	2,255 (± 88.9; 100%)	1,939 (86%)	2,170 (± 100.4; 100%)	2,494 (114%)
2022/2023	180	28	2,343 (± 88.4; 100%)	2,023 (86%)	2,212 (± 117.6; 100%)	2,372 (107%)

However, the number of ED visits on New Year’s Eve remained similar to reference Saturdays. For example, at the change of the year 2019/20, the number of cases on New Year’s Eve (1,311) was almost as high as on Saturdays (1,308 ± 127).

In contrast, there are more ED visits on New Year (1,485) than on Sundays (1,258 ± 138.4). A similar picture emerges at the following changes of the year 2020/2021 (1,445 vs. 1,235 ± 94.4), 2021/2022 (2,494 vs. 2,170 ± 100.4), and 2022/2023 (2372 vs. 2212 ± 117.6) (Table 2).

### Age distribution and risk groups

Most patients were aged ≥ 61 years. On New Year’s Eve, the proportion of this group was significantly higher than on reference Saturdays for all years (2019/2020: 41.1% vs. 35.1%; 2020/2021: 41.9% vs. 38.6%; 2021/2022: 38.3% vs. 35.1%; 2022/2023: 40.8% vs. 35.3%; all *p* < 0.001, Cohen’s w = 0.06–0.08; negligible effect).

The 21–40-year age group was overrepresented on New Year compared with reference Sundays in all years (2019/2020: 28.3% vs. 26.9%; 2020/2021: 25.8% vs. 24.3%; 2021/2022: 28.8% vs. 27.4%; 2022/2023: 28.5% vs. 26.9%; all *p* < 0.01, Cohen’s w = 0.03–0.05; negligible effect).

**Fig. 2:**
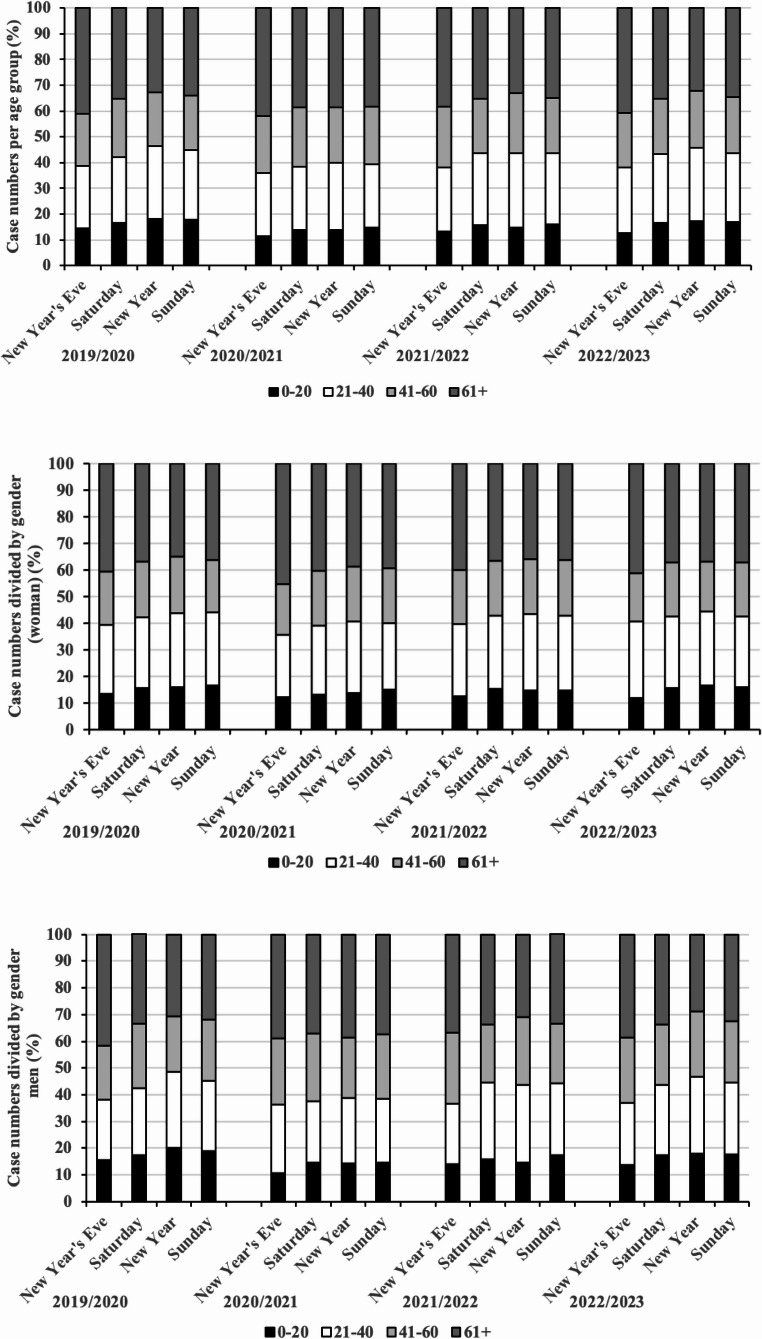
Age distribution concerning the year. (**a**) Distribution of the entire patient cohort. The > 60-year-old group presents more on New Year’s Eve, whereas the 20–40-year-old group presents more on New Year’s Eve than in the comparative periods. However, there were no differences between the females (**b**) and males (**c**) in the overall context of individual years

The proportion of patients aged 0–20 years remained stable in most years; the only significant decline occurred in 2020/2021 (14.8% vs. 16.1%; *p* = 0.02, Cohen’s w = 0.02; negligible effect) (Fig. 2a).

Age distribution patterns were comparable for both sexes, with no significant differences in male–female ratios for the overrepresentation of older patients on New Year’s Eve or younger adults on New Year (all *p* > 0.05; n. s.) (Fig. 2b and c).

### Length of stay (LOS)

**Fig. 3:**
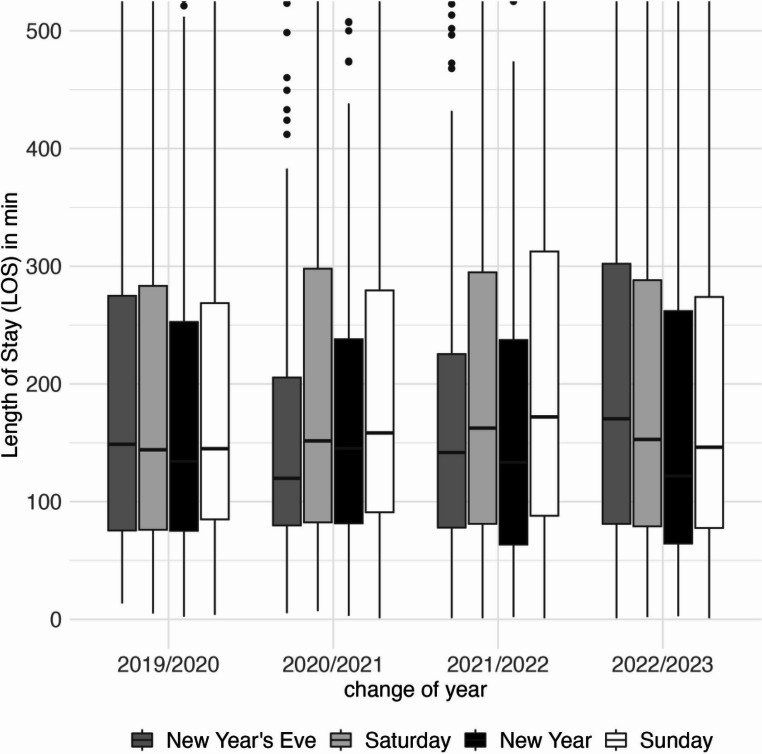
Length of Stay (LOS) during the change of years between midnight and 06:00 a.m. The Boxplots indicate the LOS of patients on New Year’s Eve and New Year. Compared with the reference period Saturday and Sunday, there are no differences. (Box, interquartile range; whiskers, minimum and maximum values)

Median LOS did not differ significantly between New Year’s Eve and reference Saturdays, nor between New Year and reference Sundays, in any year (all *p* > 0.05; n. s.) (Fig. 3).

### Injury pattern

The breakdown of the complaints presented during New Year’s Eve and New Year was first compared with the reference days of Saturday and Sunday. Compared to reference days, trauma cases declined on New Year in 2019/2020 (16.1% vs. 17.0%), 2020/2021 (15.6% vs. 17.5%), and 2022/2023 (13.1% vs. 15.6%) but slightly increased in 2021/2022 (15.2% vs. 14.7%).

Otorhinolaryngology cases were more frequent on New Year in 2019/2020 (2.9% vs. 2.8%), 2021/2022 (3.0% vs. 2.0%), and 2022/2023 (3.7% vs. 3.2%).

Ophthalmology cases remained consistent, with more eye injuries on New Year across all years.

Skin-related complaints increased each year on New Year compared to Sundays (2019/2020 (3.8% vs. 3.7%), 2021/2022 (4.5% vs. 3.1%), 2022/2023 (4.3% vs. 3.7%)), peaking in 2020/2021 (5.0% vs. 3.7%).

**Fig. 4:**
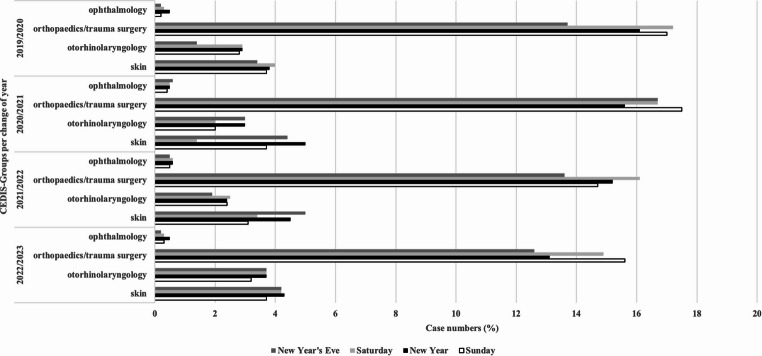
Frequency of presenting complaints. The bar chart shows the frequencies of the CEDIS-PCL main groups: ophthalmology, orthopedics/trauma surgery, otorhinolaryngology, and skin

In total we found a significant difference in the comparison of all presenting complaints over the years (Pearson’s chi-square test, *p* < 0.0001; Cohen’s W = 0.04467). Analysis of 2 × 2 contingency tables showed significantly higher proportions of orthopaedics/trauma surgery cases (*p* = 0.008; phi = 0.05) and skin-related complaints (*p* = 0.02; phi = 0.08) on New Year compared with Sundays (Fig. 4).

### Time-based subgroup analysis of CEDIS-PCL

Between midnight and 06:00 a.m. on New Year, trauma and skin-related complaints were overrepresented across all years compared with the same time window on reference Sundays. Skin-related cases peaked in the second year of the pandemic 2021/2022 (7.3%) before declining in 2022/2023 (4.5%). The number of ophthalmology patients was consistently higher on New Year than on reference Sundays in the midnight–06:00 a.m. window for all years. We also included a substance misuse category in our evaluation. Substance misuse cases decreased steadily over the years but remained higher than on reference Sundays in all years (2019/2020: 5.7% vs. 4.2%; 2020/2021: 5.3% vs. 4.1%; 2021/2022: 4.9% vs. 3.8%; 2022/2023: 3.8% vs. 3.0%).

Notably, owing to CEDIS-PCL classification, head injuries (CEDIS code 407) belong to a group of neurological disorders. Since we did not cover neurology, we added this group to otorhinolaryngology for subgroup analysis between midnight and 06:00 a.m. The combined group of neurological disorders including head injuries (CEDIS code 407) showed no differences in most years, except for a modest increase in 2022/2023 (6.2% vs. 7.2%).

**Fig. 5:**
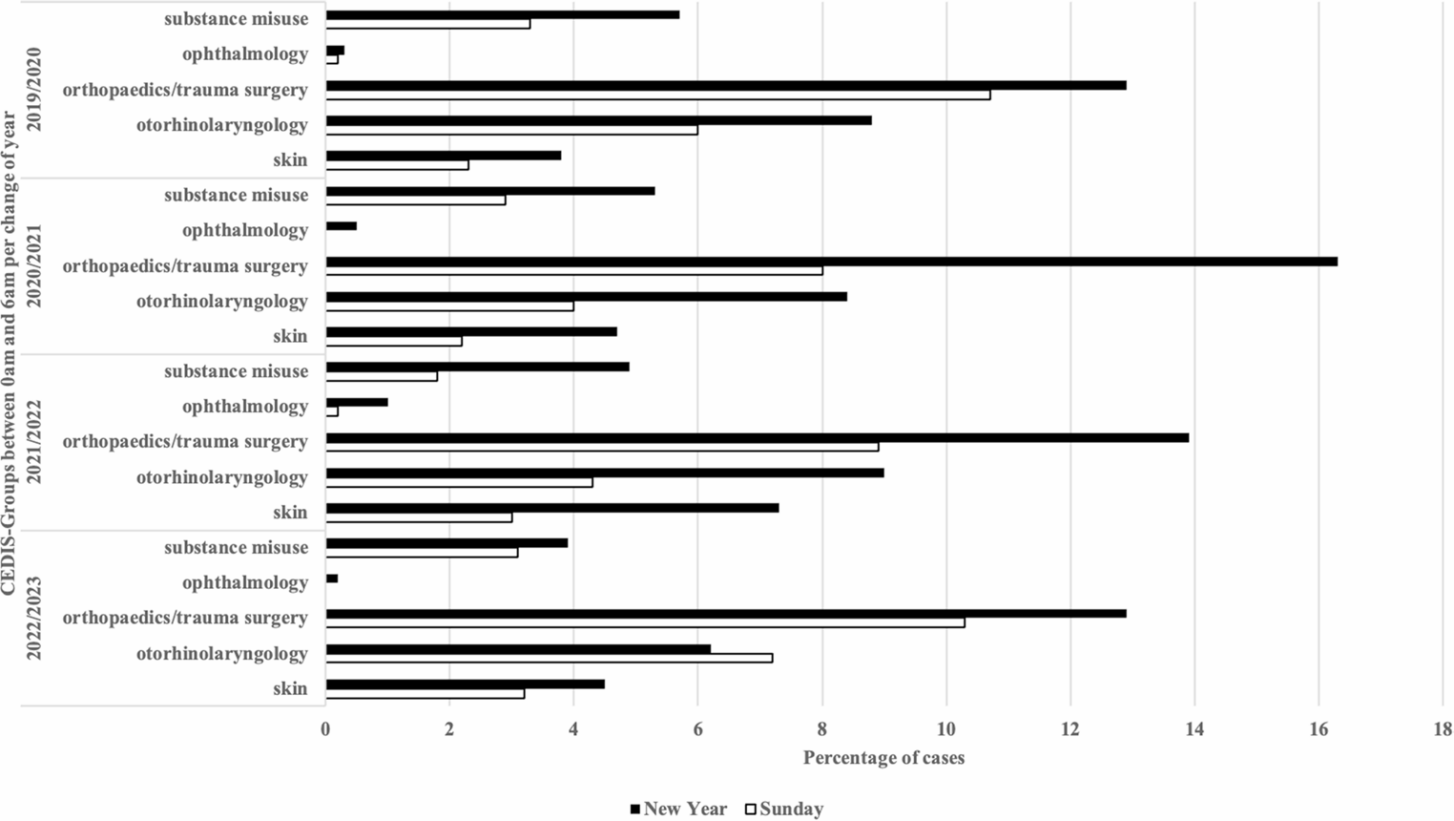
Frequency of complaints on New Year between midnight and 6:00 a.m. The bar chart shows the main groups according to CEDIS-PCL

In total, except for the CEDIS code otorhinolaryngology at the change of 2022/2023, the proportions of the individual subgroups increased compared with the reference period (Pearson’s Chi-squared test *p* < 0.0001; Cohen W = 0.1249). No significant differences were found between Sunday and New Year when broken down into the individual presenting complaints (Fig. 5).

### Time-based subgroup analysis of ICD-10

Based on the available literature, we examined the body regions most frequently injured by fireworks using the corresponding ICD-10 three-character categories. We specifically examined the injuries to the head (S00), hands (S60), and upper extremities (S40-50) between midnight and 06:00 a.m. on New Year compared with those on reference Sundays.

Head injuries (S00) were more frequent on New Year across all years (2019/2020: 0.8% vs. 0.3%; 2020/2021: 0.9% vs. 0.3%; 2021/2022: 0.7% vs. 0.2%; 2022/2023: 0.6% vs. 0.4%).

Hand injuries (S60) were also higher on New Year in all years (2019/2020: 0.6% vs. 0.1%; 2020/2021: 0.5% vs. 0.1%; 2021/2022: 0.3% vs. 0.1%; 2022/2023: 0.3% vs. 0.1%).

Upper extremity injuries (S40–S50) followed a similar pattern with higher frequencies on New Year (2019/2020: 0.4% vs. 0.1%; 2020/2021: 0.5% vs. 0.1%; 2021/2022: 0.3% vs. 0.1%; 2022/2023: 0.3% vs. 0.1%).

**Fig. 6:**
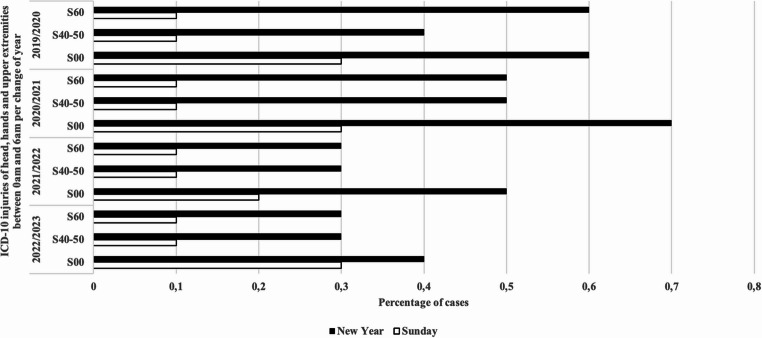
Frequencies of diagnoses (ICD-10) on New Year between midnight and 6:00 a.m. The bar chart shows the categories head injury (S00), injury of wrist and hand (S60), and injuries of arm and shoulder (S40-50) according to ICD-10

In total the percentage of the individual categories was higher than the reference period (Pearson’s Chi-squared test *p* < 0.0001; Cohen W = 0.9518). Analysis of 2 × 2 contingency tables revealed a significant increase in S00 (*p* = 0.008; phi = 0.14) when comparing New Year with Sundays(Fig. 6).

### Time-based admission frequencies

**Fig. 7:**
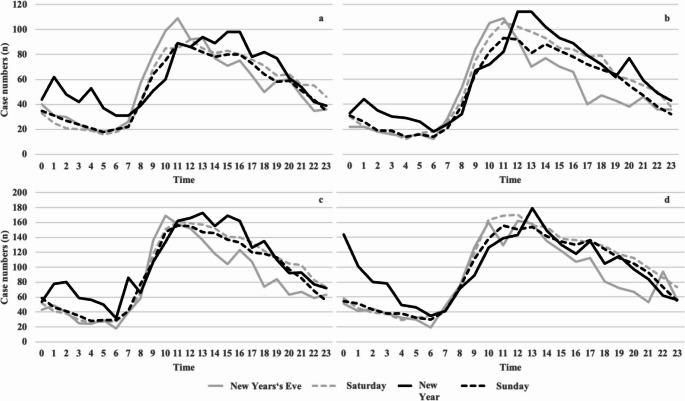
Hourly case numbers for all change of the year and reference periods. Number of cases in the change of 2019/2020 (**a**), 2020/2021 (**b**), 2021/2022 (**c**) and 2022/2023 (**d**)

The hourly calculation of case numbers showed an increase in the number of cases between midnight and 06:00 a.m. on New Year. At the chnage of 2019/2020, for example, there was an increase between 01:00 and 02:00 a.m. (62 vs. 31) and between 04:00 and 05:00 a.m. (53 vs. 21). In comparison, the number of cases declined in the following year. Nevertheless, the number of cases in New Year was also higher than in the reference period. This can also be observed during the change of 2021/2022. The sharpest increase was at the change of 2022/2023, with peaks of 144 patients between midnight and 01:00 a.m. and 101 between 01:00 and 02:00 a.m. (Fig. 7).

## Discussion

In this study, we evaluated the impact of the government’s pyrotechnic ban on ED usage rates around the turn of the year during the pandemic. Overall, the influence of the ban on case numbers and length of stay on New Year and New Year’s Eve could not be demonstrated across all scenarios. However, we observed an increase in the ED usage rate between midnight and 6:00 a.m., especially for New Year 2023, after the abolition of the corona-caused pyrotechnics ban in Germany.

This study used data from a growing registry. During the study period, several additional EDs were included in the AKTIN Emergency Department Data Registry; therefore, we compared time ranges. As New Year’s Eve is not a public holiday in Germany and generally follows a similar work and business pattern as a regular Saturday, we used the cumulative data from the following Saturdays until February 28 of the following year. Since New Year is a public holiday, we treated this constellation as a weekend and compared it with the cumulative data of the following Sundays.

Due to the corona-caused pyrotechnics ban in Germany the sale figures declined, beginning with the onset of the coronavirus pandemic. In contrast to Germany, public fireworks events were increasingly canceled in the United States during the pandemic, although no ban on private purchases was imposed. Maassel et al. reported a 55% increase in private purchases [34]. As a result, firework-related injuries increased by 47% compared with the previous year. Morrissey showed a positive correlation between increased fireworks sales and increased injuries to the upper extremities [13]. At the turn of 2022/2023, restrictions on the sale of firewood were withdrawn in Germany, increasing the pyrotechnic industry sales figures to €180 million [25]. Therefore we expected an increase in firework-related injuries as it is shown in literature. Thus, numerous studies have targeted firework-associated injuries of the eyes and otorhinolaryngology [4, 5, 7–11, 29, 35]. Some studies have indicated an increased number of patients during the turn of the year before the COVID-19-associated lockdown [4, 5]. Gabel-Pfister et al. demonstrated a significant decrease in eye injuries during the pandemic [5]. Even outside the pandemic, Faber et al. showed a reduced injury rate due to increased restrictions on the use of fireworks [19]. Nevertheless, Gabel-Pfisterer et al. showed that the number of patients increased from the first to the second year of the pandemic despite the continued ban on fireworks [5].

Framme et al. reported a significant increase in the incidence of eye injuries after the ban on fireworks [11]. We could not confirm this in our study since the percentage of eye injuries at the turn of the year remained similar (Fig. 4). There was only a slight increase on New Year compared with Sunday for each turn of the year.

However, we showed that the daily usage rate was not affected; however, between midnight and 06:00 a.m., the number of patients significantly increased by the turn of 2022/2023. This significantly increased volume can be interpreted as a possible additional burden, consistent with previous findings [11]. In contrast, we could not demonstrate the association between an additional caseload and a higher rate of complaints related to otorhinolaryngology. However, the ICD evaluation revealed a higher number of head injuries. The percentage of cases increased during the pandemic.

In contrast with other studies [5, 15, 16, 19], we did not observe an increase in the number of male patients. As we analyzed the registry data, a bias between fireworks-related injuries and injuries due to other reasons could have interfered with these results. Gabel-Pfister et al. observed an overrepresentation of eye injuries in under-aged individuals [36], especially in post-lockdown years. Our analysis of the 0–20 years old patient group showed no differences. In this context, we focused on our data regarding eye injuries as well as injuries to the head and both upper and lower limbs. Therefore, the database was slightly different, and so was the age distribution.

### Limitations

One limitation of this study is that severe individual injuries could not be distinctly identified, as they were not explicitly captured by the presenting complaints according to the CEDIS-PCL. Consequently, such cases may be obscured by background noise in the data. In this case, the documentation of cases in individual EDs could not be traced. For example, we did not determine whether a head injury is always documented as a presenting complaint or is combined with other presenting complaints. Another limitation is identifying fireworks-related injuries, which could only be accurately distinguished during New Year’s Eve and New Year by analyzing free-text entries in the presenting complaints according to the CEDIS-PCL. Seventy-five cases of fireworks-related injuries were distinctly identifiable across the four New Year’s transitions included in the study. Therefore, all the associations, trends, and observations regarding these injuries should be interpreted as preliminary or hypothetical.

Additionally, although the analyses yielded high levels of statistical significance, the effect sizes were mainly small, suggesting that the observed significance primarily resulted from the large sample size rather than a strong underlying effect. Furthermore, the AKTIN ED registry is constantly growing; therefore, the number of individual emergency admissions and the increase in the number of cases at the turn of the years differ.

## Conclusion

In this study, we showed a significant increase in injured patients between midnight and 6:00 a.m. on New Year following the abolition of the corona-related pyrotechnic ban in Germany. Therefore, a significant increase in the workload could also be concluded. This multicenter study provides novel insights into pyrotechnic-related injuries and will aid in facilitating future research. Continuing the efforts of this study, a detailed hospital level analysis of the head and severe injuries in patients admitted between midnight and 6:00 a.m in a multicenter approach are necessary for a recommendation questioning a general pyrotechnics ban.

## Data Availability

The data used in this study were obtained from the AKTIN Emergency Department Data Registry. Due to data protection regulations and patient confidentiality, the dataset is not publicly available. However, access to the aggregated data can be requested from the corresponding author.

## Declarations

### Generative AI

We declare no use of generative AI.

## References

[1] Burki TK. Are fireworks a hazard for respiratory health? Lancet Respir Med. 2017;5:103–4. 28145227 10.1016/S2213-2600(17)30016-4

[2] Galanis DJ, Koo SS, Puapong DP, Sentell T, Bronstein AC. Decrease in injuries from fireworks in Hawaii: associations with a county policy to limit access. Inj Prev. 2022;28:325–9. 10.1136/injuryprev-2021-044402. 35086916 10.1136/injuryprev-2021-044402

[3] Flockerzi V, Schick B, Bozzato A. Schlag auf schlag –Bericht über Feuerwerksbedingte knalltraumata zum Jahreswechsel 2021/2022. [Thunder and lightning-a report on firework-associated acoustic trauma at new year 2021/2022]. HNO. 2023;1–6. 10.1007/s00106-022-01260-z. 10.1007/s00106-022-01260-z PMC9969936 36847786

[4] Werz J, Greve J, Hoffmann TK, Hahn J. New year’s eve in otorhinolaryngology: a 16-year retrospective evaluation. Eur Arch Otorhinolaryngol. 2023. 10.1007/s00405-023-07966-2. 37062783 10.1007/s00405-023-07966-2 PMC10106316

[5] Gabel-Pfisterer A, Böhringer D, Agostini H. Pandemiebedingtes Verkaufsverbot von Feuerwerkskörpern in Deutschland führt Zu einer deutlichen abnahme der Augenverletzungen. [Pandemic-related sales ban of fireworks in Germany leads to a significant reduction of firework-related eye injuries]. Ophthalmologie. 2022;119:1257–66. 10.1007/s00347-022-01778-1. 36449087 10.1007/s00347-022-01778-1 PMC9713168

[6] Gabel-Pfisterer A, Böhringer D, Agostini H. Dreijahresergebnisse der Deutschlandweiten umfrage Zu augenverletzungen durch Feuerwerkskörper. [3-year results of the German nationwide survey on eye injuries caused by fireworks]. Ophthalmologe. 2019;116:1138–51. 10.1007/s00347-019-00967-9. 31659430 10.1007/s00347-019-00967-9

[7] Lenglinger MA, Zorn M, Pilger D, von Sonnleithner C, Rossel M, Salchow DJ, et al. Firework-inflicted ocular trauma in children and adults in an urban German setting. Eur J Ophthalmol. 2021;31:709–15. 10.1177/1120672120902033. 31973551 10.1177/1120672120902033 PMC8120635

[8] Chan WC, Knox FA, McGinnity FG, Sharkey JA. Serious eye and adnexal injuries from fireworks in Northern Ireland before and after lifting of the firework ban–an ophthalmology unit’s experience. Int Ophthalmol. 2004;25:167–9. 10.1007/s10792-004-1958-z. 15847316 10.1007/s10792-004-1958-z

[9] Thygesen J. Ocular injuries caused by fireworks. 25 years of experience with preventive campaigns in Denmark. Acta Ophthalmol Scand. 2000;78:1–2. 10726778

[10] Turgut F, Bograd A, Jeltsch B, Weber A, Schwarzer P, Ciotu IM, et al. Occurrence and outcome of firework-related ocular injuries in Switzerland: a descriptive retrospective study. BMC Ophthalmol. 2022;22:296. 10.1186/s12886-022-02513-9. 35799154 10.1186/s12886-022-02513-9 PMC9260982

[11] Framme C, Book B, Hufendiek K, Panidou-Marschelke E, Sinicin E, Lindziute M, et al. Spektrum von Feuerwerksverletzungen an einer Universitäts-Augenklinik Nach dem COVID-19-Lockdown. [Spectrum of firework injuries at a university eye clinic after the COVID-19 lockdown]. Ophthalmologie. 2023. 10.1007/s00347-023-01927-0. 37815541 10.1007/s00347-023-01927-0

[12] van der Zee C, Smeulders M, van de Kar A. Vuurwerkhandletsel behandeld door Plastisch chirurgen. [Hand injuries caused by fireworks and treated by plastic surgeons]. Ned Tijdschr Geneeskd. 2014;158:A8381. 25467025

[13] Morrissey PJ, Scheer RC, Shah NV, Penny GS, Avoricani A, Koehler SM. Increases in firework-related upper extremity injuries correspond to increasing firework sales: an analysis of 41,195 injuries across 10 years. J Am Acad Orthop Surg. 2021;29:e667–74. 10.5435/JAAOS-D-20-00201. 34135296 10.5435/JAAOS-D-20-00201

[14] Bitter CC, Zhang Z, Talbert AW, Weber AK, Hinyard L. Firework injuries are increasing in the United States: an analysis of the National Emergency Department Sample. JACEP Open. 2021;2:e12600. 10.1002/emp2.12600. 34918008 10.1002/emp2.12600 PMC8641913

[15] Winicki NM, Waldrop I, Orozco JV, Novak D, Sheets NW. The epidemiology of firework-related injuries in the US, 2012–2022. Inj Epidemiol. 2023;10:32. 10.1186/s40621-023-00446-5. 37403127 10.1186/s40621-023-00446-5 PMC10320921

[16] Sandvall BK, Jacobson L, Miller EA, Dodge RE, Alex Quistberg D, Rowhani-Rahbar A, et al. Fireworks type, injury pattern, and permanent impairment following severe fireworks-related injuries. Am J Emerg Med. 2017;35:1469–73. 10.1016/j.ajem.2017.04.053. 28495236 10.1016/j.ajem.2017.04.053

[17] U.S. Consumer Product Safety Commission. Fireworks-Related Injuries and Deaths Spiked During the COVID-19 Pandemic. 05.08.2025. https://www.cpsc.gov/Newsroom/News-Releases/2021/Fireworks-Related-Injuries-and-Deaths-Spiked-During-the-COVID-19-Pandemic#. Accessed 6 Aug 2025.

[18] AerzteZeitung.de. Silvesternacht: Fünf Tote und etliche Verletzte durch Unfälle mit Böllern, Angriffe auf Rettungskräf. 2025. https://www.aerztezeitung.de/Politik/Silvesternacht-Fuenf-Tote-und-etliche-Verletzte-durch-Unfaelle-mit-Boellern-Angriffe-auf-Rettungskraef-455480.html. Accessed 6 Aug 2025.

[19] de Faber JT, Kivelä TT, Gabel-Pfisterer A. Landesweite studien Aus Den Niederlanden und Finnland Zur Häufigkeit von augenverletzungen durch Feuerwerkskörper unter dem einfluss verschiedener Schutzmaßnahmen. Englische Version. [National studies from the Netherlands and Finland and the impact of regulations on incidences of fireworks-related eye injuries]. Ophthalmologe. 2019;116:1177–83. 10.1007/s00347-019-00998-2. 31776661 10.1007/s00347-019-00998-2

[20] Bundesärztekammer R. Silvesterböller dauerhaft verbieten. Bundesärztekammer. 19.12.2022.

[21] van Yperen DT, van Lieshout EMM, Dijkshoorn JN, van der Vlies CH, Verhofstad MHJ. Injuries, treatment, and impairment caused by different types of fireworks; results of a 10 year multicenter retrospective cohort study. Scand J Trauma Resusc Emerg Med. 2021;29:11. 10.1186/s13049-020-00811-z. 33413553 10.1186/s13049-020-00811-z PMC7788980

[22] Rudisill TM, Preamble K, Pilkerton C. The liberalization of fireworks legislation and its effects on firework-related injuries in West Virginia. BMC Public Health. 2020;20:137. 10.1186/s12889-020-8249-0. 32000733 10.1186/s12889-020-8249-0 PMC6993478

[23] So regeln. andere Länder privates Feuerwerk. Panorama. 02.01.2023.

[24] Fischer B, Giesel J. Wie Das Verkaufsverbot für Böller die Feuerwerksbranche bedroht. Frankfurter Allgemeine Ztg. 23.12.2021.

[25] Statista S. Umsatz mit Feuerwerk bis 2022 | Statista. 15.04.2024. https://de.statista.com/statistik/daten/studie/284913/umfrage/umsatz-der-deutschen-pyrotechnischen-industrie/. Accessed 15 Apr 2024.

[26] Bundesministerium des Inneren und für Heimat. 2020 kein Verkauf von Silvesterfeuerwerk. Pressemitteilung. 22.12.2020.

[27] Bundesministerium des Inneren und für Heimat. 2021 kein Verkauf von Silvester­feuerwerk. Pressemitteilung. 21.12.2021.

[28] Otto R, Blaschke S, Schirrmeister W, Drynda S, Walcher F, Greiner F. Length of stay as quality indicator in emergency departments: analysis of determinants in the German emergency department data registry (AKTIN registry). Intern Emerg Med. 2022;17:1199–209. 10.1007/s11739-021-02919-1. 34989969 10.1007/s11739-021-02919-1 PMC9135863

[29] Diederich LM, Pudszuhn A, Hofmann VM. Pyroverbot an Silvester 2020/2021 - Analyse von Feuerwerksbedingten verletzungen und ihrer behandlungen. Abstract- und posterband – 93. Jahresversammlung der Deutschen gesellschaft für HNO-Heilkunde, Kopf- und Hals-Chirurgie e.V., Bonn Interface - Fokus mensch Im zeitalter der technisierten medizin. Volume 5. Georg Thieme; 2022. 10.1055/s-0042-1747452. /25/2022–5/28/2022; Deutsche Messe Hannover.

[30] Gemeinsamer Bundesausschuss. Gestuftes System von Notfallstrukturen in Krankenhäusern. 2018. https://www.g-ba.de/themen/bedarfsplanung/notfallstrukturen-krankenhaeuser/. Accessed 20 Sep 2024.

[31] Brammen D, Greiner F, Dormann H, Mach C, Wrede C, Ballaschk A, et al. Lessons learned in applying the international society for pharmacoeconomics and outcomes research methodology to translating Canadian emergency department information system presenting complaints list into German. Eur J Emerg Med. 2018;25:295–9. 10.1097/MEJ.0000000000000450. 28145941 10.1097/MEJ.0000000000000450 PMC6039420

[32] Brammen D, Greiner F, Kulla M, Otto R, Schirrmeister W, Thun S, et al. Das AKTIN-Notaufnahmeregister – kontinuierlich Aktuelle Daten Aus der akutmedizin: ergebnisse des registeraufbaus und erste Datenauswertungen Aus 15 Notaufnahmen unter besonderer Berücksichtigung der Vorgaben des gemeinsamen bundesausschusses Zur Ersteinschätzung. Med Klin Intensivmed Notfmed. 2022;117(1):24–33. 10.1007/s00063-020-00764-2. 33346852 10.1007/s00063-020-00764-2 PMC7750913

[33] Johannes K, Peter D, Robert G, Kurt H, Ross I, Tomas K et al. R: The R Project for Statistical Computing.

[34] Maassel N, Saccary A, Solomon D, Stitelman D, Xu Y, Li F, et al. Firework-related injuries treated at emergency departments in the united States during the COVID-19 pandemic in 2020 compared to 2018–2019. Inj Epidemiol. 2021;8:65. 10.1186/s40621-021-00358-2. 34758871 10.1186/s40621-021-00358-2 PMC8579722

[35] Frimmel S, de Faber JT, Wubbels RJ, Kniestedt C, Paridaens D. Type, severity, management and outcome of ocular and adnexal firework-related injuries: the Rotterdam experience. Acta Ophthalmol. 2018;96(6):607–15. 10.1111/aos.13711. 29536639 10.1111/aos.13711

[36] Gabel-Pfisterer A, Lang SJ, Boehringer D, Agostini H, de Geus LC, de Faber JT. Significant increase of firework induced eye injuries in Germany and the Netherlands- are we doing enough to protect minors and bystanders? Graefes Arch Clin Exp Ophthalmol. 2024. 10.1007/s00417-024-06677-6. 39627464 10.1007/s00417-024-06677-6 PMC12095323

